# Professionals’ Expectations and Preparedness to Implement Knowledge-Based Palliative Care at Nursing Homes before an Educational Intervention: A Focus Group Interview Study

**DOI:** 10.3390/ijerph18178977

**Published:** 2021-08-26

**Authors:** Helene Åvik Persson, Gerd Ahlström, Anna Ekwall

**Affiliations:** Department of Health Sciences, Faculty of Medicine, Lund University, P.O. Box 157, SE 221 00 Lund, Sweden; gerd.ahlstrom@med.lu.se (G.A.); anna.kristensson_ekwall@med.lu.se (A.E.)

**Keywords:** educational intervention, nursing home, palliative care, attitude of health personnel

## Abstract

The provision of knowledge-based palliative care is rare in nursing homes. There are obstacles to practically performing this because it can be difficult to identify when the final stage of life begins for older persons. Educational interventions in palliative care in nursing homes are a challenge, and joint efforts are needed in an organisation, including preparedness. The aim was to explore professionals’ expectations and preparedness to implement knowledge-based palliative care in nursing homes before an educational intervention. This study has a qualitative focus group design, and a total of 48 professionals working in nursing homes were interviewed with a semi-structured interview guide. Qualitative content analysis with an inductive approach was used for the analysis. One major theme was identified: professionals were hopeful yet doubtful about the organisation’s readiness. The main categories of increased knowledge, consensus in the team, and a vision for the future illustrate the hopefulness, while insufficient resources and prioritisation illustrate the doubts about the organisation’s readiness. This study contributes valuable knowledge about professionals’ expectations and preparedness, which are essential for researchers to consider in the planning phase of an implementation study. The successful implementation of changes needs to involve strategies that circumvent the identified obstacles to organisations’ readiness.

## 1. Introduction

The global population is ageing; the United Nations [1] estimates that the number of people aged 65 or more will have doubled to 1 in 6 by 2050, from 1 in 11 in 2019. In Sweden, about 20% of the population was aged 65 years or older in 2020 [2]. It can be difficult to predict when an older person is dying because of an often prolonged period of suffering, and the illness trajectory can include both decline and improvement in condition before death [3,4,5]. The International Association for Hospice and Palliative Care (IAHPS) [6] (p. 761) definition of palliative care refers to making sure that vulnerable groups, including older persons, should have access to palliative care. The places with the highest need for palliative care are nursing homes [7]. A large proportion of the older persons who move into nursing homes suffer, alongside normal ageing, multi-morbidities that indicate a need for palliative care [7,8,9]. This will pose challenges for elderly care because delivering high-quality palliative care demands knowledge that can support the older person [10,11]. Because of the possibility of slow dying, the early introduction of a palliative care approach in a nursing home may be of importance to optimise the older person’s well-being [12,13].

The provision of palliative care in nursing homes in Sweden, as in the rest of Europe, is low [4,10]. There is a lack of support and knowledge related to palliative care for professionals in nursing homes [14,15,16,17,18,19,20]. The ability to identify when the final stages of life begin for older persons is missing to a large extent outside palliative care specialist units. The principles of palliative care need to be implemented in nursing homes, where the focus should be on both geriatric and palliative care competence since both types of expertise are needed there [3,21]. Professionals need support in dealing with ethical issues that arise in palliative care, such as the management of existential needs for the older person and conversations about death and dying [22,23,24,25]. Although expertise and knowledge are called for in nursing homes [14,15,16,17,18,26,27], there are obstacles, in practice, to delivering quality palliative care: inexperienced professionals, high staff turnover, and a lack of supervision from qualified nurses [28,29]. Research studies highlight the need to design credible educational interventions with the potential to evaluate and improve palliative care for older persons in nursing homes [30,31].

A Cochrane review from Hall et al. [16] included only two RCT studies with a focus on educational intervention in palliative care for professionals in nursing homes. It was stated that more high-quality research is needed [16]. A comprehensive integrative review concerning palliative care for adults (aged 18 or over) from Europe [32] regarding organisational changes that could improve palliative care identified implementation strategies such as educational intervention, process mapping, feedback, multi-disciplinary meetings, and mixed interventions. The educational strategies in 14 studies (two experimental and 12 quasi-experimental) included lectures, study days, role-play sessions, interactive education, educational outreach visits, and computer-facilitated education. Despite four studies showing significant improvements, the conclusion was that future research with a more rigid design is needed to identify optimal strategies to improve the organisation of palliative care in different settings, including nursing homes [32]. In two reviews [33,34] on nursing home interventions with the aim of changing professional practice, the researchers stated that palliative care intervention is complex and exploring the implementation strategies that lead to change is of great importance.

Various changes are constantly taking place in nursing home organisations. In order to implement sustainable changes in an organisation, managers and stakeholders need to make sure that the professionals involved have a positive attitude and receive the proper support [35,36,37,38]. Weiner [39] states that the professionals need to be ready if the organisational changes are to be successful. The essence of such readiness is that there should be “a shared team trait” among the members of an organisation [36,40,41]. ORC has two dimensions: change commitment, which can be described as “the collective determination of members to implement a change”, and change efficacy, which can be described as “the shared belief in their ability to do so”. Monitoring these dimensions can facilitate the organisation’s readiness to implement changes [39]. Against this background, the theory of Organisational Readiness for Change (ORC) is a starting point for designing this study.

Research on preparedness among professionals and organisational readiness for change is limited in the health care sector, and the few recent studies that are available focus on specific areas such as person-centred care in nursing homes [42], orthogeriatric care in hospital units [43], nurses’ views on hospitals’ organisational readiness for change [44], acute care at hospitals [45], long-term care [46] and residential aged-care settings [38]. The research shows that professionals have a need for information and support and the opportunity to participate in the change at an early stage, as this can otherwise negatively affect the organisation’s readiness [47]. Similarly, the attitudes and beliefs of professionals about the need for change have also been shown to have an impact on the outcome of the change [48,49]. If the professionals are well-informed about the intended change and experience support from the management, they feel more prepared for it [43,50].

The translation of knowledge into daily practice is lacking, which means there is a wide gap between the available scientific evidence and its use in practice [51]. Research shows that the implementation of palliative care in nursing homes is seen as a challenge, and educational interventions are sparse in the literature [10,32,33,34,52]. The project “Implementation of Knowledge-Based Palliative Care in Nursing Homes” (hereinafter referred to by its Swedish acronym KUPA) [37,53], of which this study is a part, can be seen as one effort to close the knowledge gap in this area. Previous studies [35,37,42,43,45,46,54] have shown that the preparedness of professionals have an influence on implementation and play an essential role when an organisation is going to make a change. However, little has been written about the professionals’ preparedness in nursing homes, and we have not found that ORC has been explored in the context of professionals’ perspective on expectations and preparedness with regard to a palliative care educational intervention in nursing homes, which makes our study unique and relevant. Therefore, the aim of this study was to explore professionals’ expectations and preparedness to implement knowledge-based palliative care in nursing homes before an educational intervention. This aim can be specified through the following two research questions:

1. What do the professionals see as the preconditions for the successful transformation of current elderly care into palliative care?
2. What do they see as facilitating such a transformation, and what do they see as hindering it?

## 2. Materials and Methods

### 2.1. Design

This study has a qualitative focus group design. The exploratory nature of this design is appropriate to use when the researcher intends to understand a new area or phenomenon [55]. Focus groups interviews were chosen because they use group interaction between the participants in order to generate the data. Another advantage is that the interaction in groups provides distinctions and a wider range of information about attitudes, knowledge, and experiences [55,56].

### 2.2. Setting

Sweden has a policy [57] of providing care to older persons in their own homes for as long as possible [58,59]. When an older person is too ill and frail to be cared for in their own home, a move to a nursing home or sheltered housing becomes necessary. In nursing homes, the right to an apartment is based on an older person’s need for everyday care, as assessed by social workers in the municipality. The nursing home provides a homelike atmosphere and offers around-the-clock care [60]. All of the professionals working in nursing homes are employed by the municipality. The most common professionals are assistant nurses (ANs). They can have one or two years of education, including gerontology, geriatrics, and palliative care; others have no training [61]. Registered nurses (RNs), occupational therapists (OTs), physiotherapists (PTs) and social workers (SWs) have at least a university degree at the Bachelor’s level.

#### The Research Setting

This study is a part of the evaluation of educational interventions in palliative care in the KUPA project, which is described in more detail elsewhere [53]. In the project, a total of 30 nursing homes were included: both larger and smaller nursing homes in both urban and rural areas in two different counties in southern Sweden [53]. The educational intervention was workplace-based and took the form of five educational seminars over the course of six months. The educational seminars conveyed knowledge and skills to be transferred into routine practice in the nursing homes and concerned five themes: the palliative approach and dignified care, next of kin, existence and dying, symptom relief, and collaborative care. They were led by two experienced clinical nurses. An educational booklet was designed as study material for the professionals and involved assignments concerning the five themes to be completed between the seminars [53].

### 2.3. Sampling and Participants

A delegated contact person at each nursing home was informed about the study and asked colleagues at their workplace about participation. The inclusion criterion for this study was professionals working in the nursing homes participating in the KUPA project [53]. Different professionals at each nursing home were selected to generate a wide range of opinions about expectations and preparedness to implement knowledge-based palliative care at the workplace [53]. This secured the sample variation in terms of staff for care, social work and rehabilitation as well as for frontline management. The credibility of the results increases when the different perspectives of the professionals in the team at the nursing home are explored. Collecting information from different professions maximises the exploration of different perspectives within a group setting [62]. The professionals represented six nursing homes of different sizes, located in different areas (rural/urban) and from both counties of the KUPA project [53]. All 48 professionals asked if they wanted to participate, all agreed of whom 45 were female and 3 male. The participants included 32 ANs, 7 RNs, 4 OTs, 2 PTs, and 3 SWs. One-third of the participants had a Bachelor’s degree. Further background data were not collected.

### 2.4. Data Collection

The participants were interviewed in 6 focus groups, with 6–10 included in each group, directly before the educational intervention started in the two counties. All of the interviews took place in the nursing homes where the professionals were working. The focus group interviews were led by a researcher as a moderator (M) with experience in leading focus groups, whilst a second researcher acted as assistant moderator (AM), observing the interviews, taking notes and monitoring the recording equipment. The interviews started with the moderator explaining the purpose of the interview and asking questions based on the semi-structured interview guide, which was developed for a study on ORC theory [39].

The semi-structured interview guide included the following five broad questions and probing follow-up questions, depending on the discussion:

1. What does the educational intervention in palliative care mean for the nursing home in which you work?
2. What are your thoughts on how palliative care education will be carried out after the KUPA project at your respective nursing home?
3. How do you currently assess the readiness of your organisation for change?
4. How do you view your managers’ readiness to change (i.e., to motivate and engage)?
5. In your opinion, what facilitates or complicates the implementation of palliative care?

Face validity was applied. Three geriatric and palliative care researchers examined and assessed the formulation of the questions in the interview guide regarding understanding, relevance to the aim of the study and clarity. The review resulted in some minor clarification in the wording of the questions. At the start of the focus group, everyone introduced themselves. The focus group interviews had an average duration of 38 min. All digitally recorded interviews were transcribed verbatim.

### 2.5. Data Analysis

The analysis of the interview text involved qualitative content analysis with an inductive approach, i.e., going from empirical data to abstractions and generations of categories and themes [63]. The analysis was conducted by the first author (H.Å.-P.), who read each interview several times to obtain an overview of the content. Thereafter, meaning units (annotations) were identified and then condensed and labelled with a code (node). Annotations can be notations that are linked from a chosen segment in the text, while coding brings data together and is the most central and important function within qualitative data analysis [64,65]. Through the codes (nodes), subcategories were generated and sorted under higher-order headings (a structured hierarchy was constructed with different levels of under-nodes). Next, categories with similar content were grouped (creating new, more comprehensive nodes), which resulted in the main categories being generated and a theme arising.

The co-authors (G.A., A.E.) read the transcribed interviews and independently reviewed the subcategories, categories and themes. The interpretation of the findings was discussed by all three authors, and this process lasted until a satisfactory interpretation was reached. Trustworthiness was upheld by means of investigator triangulation involving several researchers [55,65], which significantly enhanced the credibility of the findings. Examples of the analytical procedure are given in Table 1. For the analysis, the 12th version of NVIVO software [64] was used.

## 3. Results

**Figure 1:**
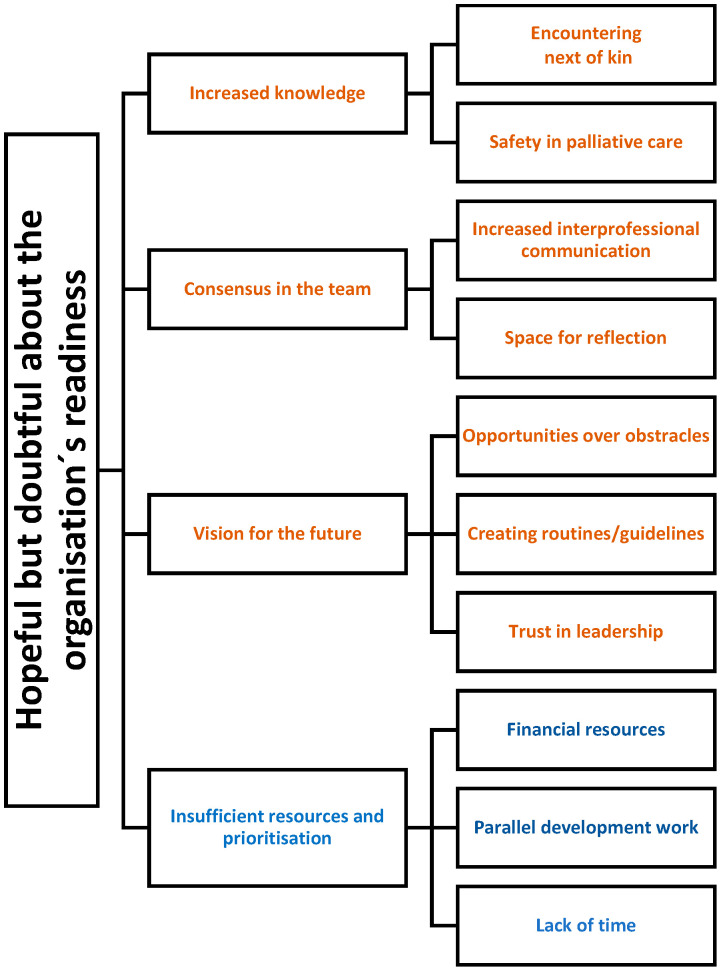
The results, as described in a theme, main categories, and subcategories. The main categories in brown are connected with hope and those in blue with doubts about the organisation’s readiness.

The professionals indicated that palliative care starts well before the older person’s last days and can, in fact, be a long process that begins the day the older person moves into a nursing home. The participants emphasised that it was important for them to gain knowledge about palliative care so that they can start this care as soon as the older person is informed about their incurable health condition. Participants believed that care during the whole dying process should be the focus, not just the last days in life, which is most common today. However, the participants emphasised that palliative care was a difficult task for them. The results consist of 1 theme, 4 main categories, and 2–3 subcategories in each main category (Figure 1).

### 3.1. Hopeful but Doubtful about the Organisation’s Readiness

The theme “hopeful but doubtful about the organisation’s readiness” describes the overall message in two dimensions. The participants experienced hopefulness but voiced a lack of trust regarding the preparedness of the organisation concerning changes to palliative care. The participants emphasised several times that palliative care is an important and prioritised field. They were hopeful about what educational intervention could achieve for both themselves and their workplace. However, the participants were sceptical of the organisation’s readiness to implement palliative care in everyday work after the educational intervention. For such a change to be possible, plans must be drawn up and implemented. These limitations in preparedness contributed to the participants’ doubts. Despite the doubts, the participants expressed hope that this education would lead to something positive and highlight the importance of improving the care they provide.

### 3.2. Increased Knowledge

The participants had expectations of gaining increased knowledge about palliative care through the educational intervention. Overall, the perception was that knowledge about palliative care needs to be updated at regular intervals, as the participants believed that palliative care requires greater attention in the workplace. At present, this continuous acquisition of knowledge was lacking, which contributed to the participants feeling that this educational intervention was especially important. Gaining the opportunity for increased knowledge in palliative care was considered valuable by the participants, both for creating safety and for increasing the quality of care in the final stages of life for the older person, as the below excerpt from an interview transcript indicates.

PT: I see it as a chance to learn more.
AN1: More knowledge.
PT: Deepen the knowledge, yes exactly.
AN2: I want to refresh mine because I’ve done palliative specialist education, but it’s a couple of years ago now.

#### 3.2.1. Encountering Next of Kin

The participants expressed that palliative care is often emotionally demanding, such as meeting with the next of kin who are in crisis. The participants expressed a desire to obtain more knowledge and support about how to encounter next of kin in palliative care. Their uncertainty was about how involved they should let the next of kin be and how they should respond to next of kin who are worried, disagree with family members, or feel guilty. Uncertainty also emerged about the treatment of the next of kin after the older person’s death. The participants emphasised that it is not only the older person who is having a difficult time at the end of life but also the close family members. As a result, they were hopeful of gaining more knowledge about encountering next of kin.

#### 3.2.2. Safety in Palliative Care

The participants emphasised a wish to gain more knowledge about palliative care to ward off fears within themselves as they have difficulty with death, sometimes due to personal experiences. They wear a “mask” when caring for older persons to be able to handle the situation. In palliative care, the ANs stressed that safety is ensured when the RN contacts the ANs to check the status and follow-up of the given medicine. With more knowledge, the ANs expect to be able to act as mentors for new colleagues, support each other, and feel secure in their professional role. Furthermore, the participants believed that, since palliative care is so difficult, working with professionals who have competence in palliative care is better than working with colleagues who have no knowledge, which was stated as relatively common.

### 3.3. Consensus in the Team

The participants felt hopeful that the educational intervention would create a consensus in the care team and lead to an increased understanding of each other’s roles. Often the focus is on one’s own work, and the understanding of others’ professional roles may become less clear, which can lead to poorer collaboration. One example that the participants highlighted was the importance of a consensus between the manager and employees. It benefits everyone in the organisation if the manager and employees have understanding and respect for each other’s knowledge and roles, such that problematic situations can be raised and discussed. They emphasised the importance of striving towards the same goal and everyone working equally to achieve the goal. The participants believed that speaking the “same language” can obviate difficulties. To provide palliative care of good quality, a consensus within the team, where everyone works together, is an important piece of the puzzle.

OT: Maybe new knowledge, I think. And maybe a consensus in the team?
RN: Yes, exactly.
OT: That we can … understand each other’s roles and maybe get more knowledge about what to do.
OT2: And have an opportunity to raise these issues.
OT1: That we work equally … yes, exactly.
AN1: That it should not be different if you live in House 6 or House 5.
AN2: No.
AN1: Or where you live, the working procedure should be the same … because I think it is different today. Or so I imagine, anyway.
MA: Yes, the perceptions differ.

#### 3.3.1. Increased Interprofessional Communication

The participants expressed the hope of being able to create more sustainable communication in the working group with the help of the educational intervention. At the moment, the professionals do things differently on different occasions and without an established consensus. As the goal is to provide the best care possible, the majority of the participants feel that communication should be improved to reduce the gap between professionals. For example, contact with the primary health care physician (GP) is something that the participants feel is lacking. The ANs emphasised that they work most closely with the older person and can convey valuable information to the GP and want to be included in his/her meeting with the RNs. Another aspect that is underlined is that GPs seldom have “end of life discussions” with older persons and their next of kin, which results in difficulties for the professionals who are charged with planning the older person’s last days. Furthermore, the ANs hoped for better communication with the RNs on how to perform palliative care. They stressed that it would often be easier if they had received more background information about the older person from the RN or had access to the medical records. This knowledge would save some calls to the RN if, for example, the older person has a history of a certain behaviour. The participants were hopeful of achieving increased communication in connection with this educational intervention, as different professions will be gathered and can discuss different points of view.

#### 3.3.2. Space for Reflection

Participants were hopeful about the opportunity to reflect with the whole team through this educational intervention and to be able to discuss and exchange experiences. In many cases, the work goes on without the opportunity for reflection together within the team, for example, after a person’s death. The participants stated that it can feel good to talk to the whole team when there are questions from the next of kin or demanding situations involving the older person who is dying. The ANs emphasised that they feel left out as they often work on their own in the ward without an RN on-site and that time spent in reflection together provides emotional relief. The participants said that sometimes many deaths occur within a short period of time, and the work just goes on without a chance to stop and reflect. The participants underlined that there is a great need to reflect with all professionals and that the day and night professionals are rarely given the opportunity to reflect together.

### 3.4. Vision for the Future

Most of the participants already had a plan for what would happen after they completed this educational intervention. Further educational sessions will be started with colleagues in the workplace so that everyone has the opportunity to attend. All new knowledge must be disseminated to colleagues like “ripples in a pond” so that the knowledge does not stay within the original group. Some of the participants called this a living project that should continue after the educational intervention is completed. Other participants had plans to deliver education for half or full days; for example, they have planning days, which means that everyone has the opportunity to participate and no one needs to be absent due to a lack of professional resources. Some participants had a vision for creating short-term palliative places in nursing homes, where older persons spend their last days. Another plan was that the participants could have a consulting role that supports palliative situations around the municipality. For example, if there are professionals who are unsure about something concerning palliative care in the home care service, the consultants can support with palliative expertise.

M: Do you know if there is a plan for how the introduction of palliative care will be carried out after these educational seminars?
AN1: Yes, we will have training of professionals … in groups.
PT: Yes.
AN2: As study circles or group discussions.
AN1: I think it is meant that me and two others should perform them.

#### 3.4.1. Opportunities over Obstacles

The participants stated that having an interest is essential for the educational intervention to be successful. Having the opportunity to learn something new is seen as positive, and the participants stressed “opportunities over obstacles.” Some RNs felt that it is easier for them to implement changes in the workplace or introduce routines, as they felt that they were more prepared due to their nursing work. The ANs agreed with this reasoning and considered that it was a great advantage for the RNs to also attend this educational intervention in palliative care. They noted that it could then be easier to influence the working group, as a colleague with higher education can have a greater influence. In general, the participants saw it as an exciting challenge to implement palliative care into the working group after the educational intervention.

#### 3.4.2. Creating Routines/Guidelines

The participants stated that there are no local routines regarding palliative care. The participants believed that having routines facilitates work and can contribute to everyone becoming equally skilled in palliative care at the workplace. The educational intervention should create a foundation of knowledge that can be used to create local routines for the workplaces. Visions of creating routines for palliative care that can be applied to all nursing homes in the entire municipality were highlighted. Some workplaces have checklists containing practical items, such as white sheets, but the participants considered these old-fashioned as there are now more options for what to cover the body with after the death. The participants also wanted to create routines for how the other residents of a nursing home should be informed after someone’s death. Another fact that was emphasised is that some older persons are of foreign ancestry, which makes it difficult for the participants to know what cultural traditions are expected before, during, and after death. In these cases, routines are also called for. Some participants had a hope of being able to create a “palliative folder” that will be available to everyone in the workplace. In that folder, there must be guidelines for procedures in palliative situations; for example, there must always be a professional present when death is expected to occur shortly.

#### 3.4.3. Trust in Leadership

The participants highlighted leadership as being of great importance for preparedness when changes are to be made in an organisation. There is a need for leadership that structures the conditions so that palliative care can be implemented in the organisation, and, overall, managers were perceived as being positive towards change and development. The participants believed that education in palliative care is important for professionals and for the workplace and is seen as a priority area that is shared with the managers. The participants felt that the ANs’ managers are more involved in the changes that take place and in the education that is given because the manager is usually on-site at the nursing home. The majority of the managers, even those new to their positions, actively try to increase their employees’ professional competence and, thus, considered this education regarding palliative care as important to attend. The participants saw rewards from different points of view, and, for some, it meant the opportunity to gain education and knowledge.

### 3.5. Insufficient Resources and Prioritisation

An imminent problem that the participants pointed out was a lack of personnel resources, which limits the possibility of implementing palliative care in the workplace. Even if the personnel unit has a list of standbys, it is rare for replacements to be obtained. Most of the standbys on the replacement list say no to work. Managers rarely say no if an AN wants to take time off to attend a course, but the replacements hired are often inexperienced, which makes it difficult for regular workers as they have to support and supervise the substitutes. The use of existing resources and covering for each other are the “guiding stars” in the organisation. However, this tends to mean that there is limited room for professional development as education is pushed into the rest of the work. The RNs confirmed that there is a problem with a lack of standbys, which limits the opportunity to attend courses. This lack of professional resources is experienced by the participants as stressful, and a feeling of inadequacy can arise. The participants stressed that they ’turn themselves inside out’ to solve the professional shortage and sometimes feel frustrated about the situation. There was also a general perception that sick leave in the workplace has increased recently. Another general view was that decisions taken higher up in the organisation do not reach the professionals until long after the decision has been made, even if it concerns the goals for the organisation.

AN1: It is not just the will; there must also be resources.
RN: And the message is that you have to work with existing resources.
AN1: Yes, yes.
RN: So you have to try to push it into the ordinary work schedule.
AN2: And do much, much more at the same time.

#### 3.5.1. Financial Resources

The participants stated that financial resources are a limitation for implementing palliative care in the workplace. It was well known among the participants that there is a tight budget, and it is often economics that controls change decisions. The participants stated that they often receive a message from the management stating that there is a lack of money to employ more professionals. They expressed their dissatisfaction with the fact that it is often impossible to have an extra professional present when death is expected to occur shortly. The participants considered this to be regrettable, as economics should not be allowed to limit palliative care; resources really need to be invested. Sometimes they have to solve this situation themselves with frequent inspection visits instead of a professional being present with the older person. The participants emphasised that economics is not something they have insight into or can directly influence. However, they try to solve different situations in the best possible way in the workplace.

#### 3.5.2. Parallel Development Work

The participants stated that there were various projects going on in the workplaces that can limit the implementation of palliative care. Several parallel development works were perceived as being too much to take in at once, and difficulties with making use of the knowledge were underlined. These development projects should have been spread out over a longer period of time. Some of the participants had asked their managers which project they could set aside in order to participate in the planned educational intervention in palliative care. These projects must also be “squeezed in” between ordinary work tasks, which can be stressful for the participants. Development projects that ran in parallel for the participants at the start of this educational intervention in palliative care concerned areas such as dementia, documentation, quality registers, nutrition, mental illness in older persons, and older persons’ health and quality of life. The participants described feeling as if “there is a lot on our shoulders now”, but, at the same time, they were curious and expectant about the educational intervention in palliative care as this was perceived as a high-priority area.

#### 3.5.3. Lack of Time

A clear limitation on the educational intervention in palliative care at the workplace was time. The participants believed that, in addition to all their other tasks, they needed to make time to attend the educational intervention. Time always plays a major role and creates stress among the participants, who hoped that there would be no clashes with their practical tasks. The participants talked about times when they were stressed when caring for dying persons, when they knew that their colleagues needed help out on the ward. Furthermore, the participants stressed that there are many tasks that they have to manage in addition to nursing efforts, such as paperwork, cooking supper, and cleaning, which are time-consuming. The participants emphasised that they experience difficulties in sitting down in peace and quiet to complete paperwork as they are often interrupted by, for example, colleagues who need help.

## 4. Discussion

The findings show that the professionals had hopes but also doubts regarding the preparedness of the organisation to implement the necessary changes in palliative care. The professionals had expectations that the educational intervention could contribute to increased knowledge and make the remaining time for older persons as pleasant as possible. Another benefit from the professionals’ point of view was consensus in the team, which includes a common view of palliative care so that everyone in the team works in the same way. Furthermore, increased interprofessional communication between the professions was underlined in the discussions. The last expectation included visions for the future that represent the participants’ forthcoming plans, i.e., a desire to create routines that improve palliative care. The doubts about the preparedness of the organisation were concerned with insufficient resources and prioritisation, which include finances, other professional development work, and time. As the competence profiles among the participants varied greatly, different aspects specific to different professional groups were reported.

The participants’ rated their managers as being positive about development within the organisation and also the educational intervention in palliative care. Furthermore, the participants expressed that managers have an essential role to play when a change is going to be implemented in an organisation. The fact that the manager has an important role to play in improving palliative care in nursing homes was also identified in other studies [66,67]. In a study by Nielsen et al. [37], leadership was not a barrier to developing evidence-based palliative care. The readiness of the organisation includes requirements for leadership [39]. The manager must explain the importance of the implementation and encourage employees to be motivated and committed. It can be a challenge getting professionals to support a proposed change because, most often, a change in the wider nursing home culture is needed [68,69]. Nielsen et al. [37] stressed that when palliative care principles from cancer care are transferred to more general settings, i.e., nursing homes, a combination of knowledge and a change in culture (such as norms and values) is needed. Froggatt [68] also stated that a combination of a change in culture and education may achieve better improvements in nursing homes. The participants in this study had several development projects underway alongside this educational intervention. Regardless, motivation and a positive attitude towards change were predominant, which may largely be due to leadership, as leaders’ guiding principles are passed on to professionals in order to help them understand what must be prioritised in the workplace.

The participants hoped to gain increased knowledge about palliative care through this educational intervention. A lack of updated knowledge and a feeling of ignorance in the field compared to other professionals were also seen in previous research [27]. The provision of palliative care to older persons requires both geriatric and palliative expertise. The Swedish “ageing in place” ideology [57] stipulates that the older person should be able to live in their home as long as possible. This has meant that it is only the frailest older persons who move into nursing homes near the end of life, which results in their survival being relatively short [19,70]. This requires preparedness from the professionals to address complex care needs when older persons move into nursing homes. Research has found difficulties for professionals in nursing homes in terms of identifying the early signs that precede dying and determining when a person’s end-of-life stage has begun [12,71]. It is also stated that a palliative care approach needs to be implemented early in the process, right when the older person moves into the nursing home [12]. The IAHPC definition [6] (p. 761) of palliative care embraces a focus on education, i.e., general palliative care can be provided by professionals with basic education in palliative care. The National Health and Welfare guidelines [72] underline that professionals working in palliative care need continuous support and training. To summarise, our results show that professionals’ expectation of increased knowledge of palliative care might enable them to improve palliative care according to the older persons’ needs, alongside their quality of life.

Another finding of this study points to the need for better communication among professionals in nursing homes. The majority of the participants felt that communication should be improved. Communication and relationships form one of the four essential parts of palliative care; the remaining three cornerstones in palliative care are symptom relief, multi-professional collaboration, and support for next of kin [13]. Research shows several barriers to communication between professionals, such as fear of harming relationships with colleagues, worry about others disapproving of one’s opinion, and hierarchical barriers. Furthermore, professionals can also remain silent because of fear of negative attitudes from those colleagues with higher status [73,74,75,76,77]. In the context of nursing homes, where the professionals work as a team, it is essential to identify and eliminate barriers in order to achieve open and tolerant communication in the workplace. The participants also highlighted a need for reflection within the team, which could be one way to improve communication. It is likely that the understanding of each other’s role in the team increases when there is an opportunity to reflect together. This is in line with existing research that states that, given the opportunity to reflect on past performance, interactions and decisions, team members can share and update thoughts and feelings, which can further improve their performance [78]. Reflection can be a tool to incorporate experiences into learning and help others examine their attitudes, beliefs, and actions and then make conclusions that lead to further learning [79]. According to our results, continuously creating space for reflection, regardless of whether an incident has happened or not, may be a precursor for improvement in the workplace, promoting clear communication between professionals. The participants were hopeful that this implementation could make such an improvement possible.

The participants also highlighted obstacles they experience in the implementation of the educational intervention, such as time, money, and resources. This is in line with managers’ views of the barriers to developing evidence-based palliative care in nursing homes [37]. According to Weiner [39], these aspects are linked to the context in a way that affects the organisation’s members’ readiness to implement change. Overall, the participants in this study saw more opportunities than obstacles to implementing knowledge-based palliative care in nursing homes. Weiner [39] underlined that organisational readiness is connected to members’ commitment to change and efficacy at implementing organisational change. Change commitment can be defined as how the members of the organisation value a specific change, while change efficacy focuses on the organisational members’ shared beliefs in their collective capabilities [39]. In exploring the professionals’ expectations and preparedness before an educational intervention, it could be helpful to examine whether both change commitment and change efficacy exist in the members of the organisation. This reflects the fact that professionals mainly have an optimistic view and so their expectations about the educational intervention are hopeful. However, there was also scepticism about the organisation’s preparedness to implement this educational intervention, which may be an indication that readiness for change is perceived as not fully existing within the organisation. If a professional is not motivated or has given up, it will be an even greater obstacle to implementation. The results of this study are useful for further research exploring whether an intended implementation is the right choice and helping members of an organisation adapt, depending on their expectations and preparedness. This becomes an important societal issue when a change is going to be implemented in an organisation. Our results may also be of value in the planning phase of an implementation study because the professionals’ views are highlighted in terms of both hopefulness and doubts, and the planning can be designed accordingly.

### Limitations and Strengths

In this study, we used the focus group method to gather data on professionals’ expectations and the preparedness of the group as a whole at six nursing homes. Focus groups represent a considerable organisational effort and expenditure of time [62]. However, an advantage of focus groups is that the group process helps participants to identify and clarify their views [56], and identification and prioritisation of important topics and opinions are possible [62]. In this study, the focus group interviews were performed right before the educational intervention in palliative care began. In that way, the participant was already in place, and an extra meeting for the interview was not necessary. However, another aspect that needs to be highlighted is the role of the moderator and co-moderator in the focus group interviews and educational intervention. The moderator led the education session right after the focus group interviews, and the assistant moderator attended five seminars at one of the six nursing homes. These researchers were not known to the professionals, and socially desirable answers were not identified in the analysis of the focus group interviews. The analysis of the study was conducted independently (not by the leader of the educational intervention), which was intended to make the approach as open-minded and critical as possible in order to minimise the influence on the results.

In this study, quotations from the participants were used to strengthen credibility. To further strengthen credibility, the first author’s initial analysis of the data was read by the other authors (G.A., A.E.), and regular meetings were held throughout the analytical process for discussion and reflection upon the findings. A way to increase the dependability was that several researchers were involved in the analysis process and contributed with different interpretations until a consensus was reached. This is in line with Sandelowski [80], who stated that one way to address dependability is to include several researchers in the analysis process.

The focus group interviews included participants from six nursing homes in two different counties in Sweden. The study was conducted in Sweden and represented professionals involved in Swedish elderly care. In other countries, there may be other professionals and structures in similar organisations. Generalisation is not the goal in qualitative studies but, rather, transferring the results to another context [81]. In Section 2.2, the research setting was described; more detail can be found in the study protocol of KUPA [53]. The reader who wishes to transfer the results to a different context is responsible for making an assessment of how sensible the transfer is [81].

A limitation is that complete background data were not collected in one of the counties, which means that the individual variation is not known. This means that only information about the participants’ gender and profession was available. What could have added more to the background data is information about the age of the participants, which would have given a clearer picture of who the participants were.

Some questions in the interviews sometimes needed to be repeated in order for the participants to be able to give an answer. It may be that the participants did not fully understand the question and wanted to hear it again. It may also be the case that the participants had not participated in a research study before and were, therefore, uncertain about various concepts. In some cases, the moderator and the co-moderator clarified what was meant by the questions. However, answers to all questions were obtained after the necessary repetitions, and the experience was that the participants understood the purpose and content of the questions.

One strength of the method was that there were 6–10 participants in each focus group. A focus group usually consists of up to 12 participants [55,56], not too large a group, which means that all participants can express their point of view and that the group dynamic functions well. It is also a strength that a total of 48 participants participated in the focus group interviews, which enriched the variance in the data. The fact that the discussion can veer away from the research question is a known disadvantage of focus groups [80]. According to Kitzinger [56], it is important to be aware of the hierarchy in a focus group that contains a mix of professions. The moderator monitored the interviews so that the discussions did not go off in unintended directions; if they did so, the participants were led back to the right track, and follow-up questions were addressed to those who were less talkative.

The dissemination of the findings of this study increases the understanding of the significance of preparedness within an organisation when change is to be implemented, not least when it comes to a change involving an increase in the availability of palliative care, where knowledge is a central component. Implementing knowledge in palliative care is complex, and a thorough consideration of potential barriers and facilitators is called for. In particular, the professionals’ attitudes and preparedness need to be taken into consideration in the planning and design of implementation strategies and follow-ups to monitor the level of success.

## 5. Conclusions

The study shows that professionals’ expectations with regard to the training in palliative care are characterised mainly by the hope of increased competence and an improved work climate. However, they were sceptical about the organisation’s readiness as it requires a lot from the organisation in resources and prioritisation. The findings concerning professionals’ expectations and preparedness before an educational intervention offer guidance to managers in the planning of implementation in clinical practice and for researchers in designing an implementation study. The knowledge of an organisation’s readiness enables the successful implementation of an intervention through process mapping, feedback and multi-disciplinary meetings.

## Figures and Tables

**Table 1:** Examples of the analytical procedure.

Meaning Unit	Condensed Meaning Unit	Code	Sub- Category	Category	Theme
I know myself when I worked in a nursing home, I thought that, like, ugh, everyone just dies. There are periods when you think, oh, now so many ... so many have passed away.... Well, it affects how you work, of course. But still, you need to be able to feel that we’re doing a good job. And it’s like you say, we need to stop and reflect. But there’s rarely a chance to do that.	Sometimes I thought that everyone just dies. There are periods when you think: so many.... It affects how you work and how you feel as to whether we’re doing a good job. Rarely a chance to stop and reflect.	Create space for reflection	Space for reflection	Consensus in the team	Hopeful but doubtful about the organisation’s readiness
					
No, I don’t think it’s a sensitive question, but it’s one that’s difficult to answer because we’re all the time being fed with the notion that it’s up to us, it’s a question of the budget, that’s the way things are, we only get this, there’s not the money to employ more professionals. That’s the answer you get.	Tight budget with a limited amount of money. Not enough money to employ more professionals.	Limited financial resources	Financial resources	Insufficient resources and prioritisation	Hopeful but doubtful about the organisation’s readiness

## Data Availability

The datasets used and analysed during the study are available from the project leader (G.A.) upon written request and in accordance with ethical approval.
